# A 6-month follow-up for patients who underwent Accelerated Epi-on Collagen Crosslinking

**DOI:** 10.22336/rjo.2024.77

**Published:** 2024

**Authors:** Alina-Cristina Chiraples, Diana Maria Darabus, Horia Tudor Stanca, Mihnea Munteanu

**Affiliations:** 1 Department of Ophthalmology, “Victor Babeş” University of Medicine and Pharmacy, Timişoara, Romania; 2 Department of Ophthalmology, “Carol Davila” University of Medicine and Pharmacy, Bucharest, Romania

**Keywords:** accelerated epi-on crosslinking, visual acuity, topography, A-CXL = Accelerated collagen crosslinking, CCT = central corneal thickness, TCT- thinnest corneal thickness, UCVA = uncorrected visual acuity, BCVA = best corrected visual acuity

## Abstract

**Purpose:**

Accelerated Collagen Crosslinking (A-CXL) is widely used worldwide as an effective treatment for keratoconus due to its efficiency and rapidity. This paper aims to identify relevant changes in visual acuity parameters and topographic measurements before and six months after treatment.

**Methods:**

A total of 30 eyes from 20 patients who underwent A-CXL between May 2021 and June 2023 and had at least six months of follow-up were analyzed retrospectively. Comparisons between uncorrected visual acuity, best corrected visual acuity, spherical and cylinder diopters, and topographic measurements like Kmax, SIf, SIb, KVf, KVb, BCVf, BCVb, TCT, and CCT were made before and six months after surgery.

**Results:**

After the accelerated Collagen crosslinking (A-CXL), BCVA improved, cylinder diopters and Kmax values were reduced, and central corneal thickness (CCT) and thinnest corneal thickness (TCT) were elevated.

**Discussion:**

The study has shown results comparable with other studies regarding the heterogeneity of the group study, good outcomes in measuring the corneal central thickness and thinnest corneal thickness, and good evolution of the refraction and visual acuity. Regarding the specific topography markers, like SIf/SIb, KVf/KVb, BCVf/BCVb, and K_max_, minor differences were observed at 6 months follow-up and were not considered statistically relevant. Other studies show a better correlation at 1-year check-ups and similar results at 6-month follow-ups.

**Conclusion:**

This method’s main benefits are its brief duration and minimum intrusion, improvement of visual acuity, and the possibility of reintervention for better outcomes.

## Introduction

Like many other medical terms, the origin of keratoconus dates to ancient Greek, from the words “keras” and “conus”, meaning cone-shaped cornea [1]. Keratoconus is considered a bilateral and asymmetric ocular disease that results in progressive thinning and steepening of the cornea, leading to irregular astigmatism and decreased visual acuity [2]. The theory that states its non-inflammatory nature tends to be obsolete, and several studies have reported associations with significant alterations in inflammatory mediators, indicating that keratoconus eyes often experience some form of ocular inflammation [3]. Without being a widespread pathology, it is usually identified in patients with myopic astigmatism, who address the ophthalmologic clinics for laser refractive surgery. Based on numerous comparison studies, most common in Asian and Middle Eastern populations, it appears to correlate its frequency with race rather than geographical location [3,4].

Since 1854, when the British ophthalmologist John Nottingham wrote the first paper about the keratoconus entitled “Practical Observations on Conical Cornea, and the Short Sight and other Defects of Vision connected with it”, the methods used in diagnosing this disease changed dramatically. However, many of John Nottingham’s notes and observations from his study are still accurate [5]. In the last 170 years, mild cases of keratoconus have been treated with different optical devices, and surgery is regarded as a last resort for severe cases [5]. Nowadays, we use glasses, soft contact lenses, hard contact lenses, corneal crosslinking, corneal ring implants, and keratoplasty to stabilize, treat, and overcome the symptoms of keratoconus.

Between all these treatment methods, crosslinking of the corneal collagen has been studied, improved, and adapted to have better outcomes and minimal drawbacks. The standard procedure described in Dresden Protocol for crosslinking consists of numbing the eye surface with anesthetic drops, removing the corneal epithelium, applying a riboflavin solution (0.1% riboflavin-5-phosphate and 20% dextran T-500) to the corneal surface for 30 minutes and exposing the cornea to 370 nm UVA with an irradiance of 3 mWcm^-2^ for 30 minutes, during which time riboflavin is re-applied at 5-minute intervals [6]. Although highly efficient, this method has shortcomings: de-epithelization of the cornea can determine intense ocular pain, and the length of the procedure can generate low patient compliance, especially among children. This study is based on the accelerated epithelium-on corneal crosslinking in its attempt to overcome those disadvantages. The study aims to evaluate the changes in visual and topographic parameters.

## Methods

Thirty eyes were submitted for the data study from 20 patients who underwent Accelerated epi-on crosslinking (A-CXL) in Prof. Munteanu Ophthalmologic Center (Part of “Victor Babeş” University of Medicine and Pharmacy) in Timişoara between May 2021 and June 2023 and had at least six months of follow up. Underaged (less than 18) patients missing their six-month appointment and those using other surgical treatment methods were excluded from the study. The study was endorsed by the ethical committee of “Victor Babeş” University of Medicine and Pharmacy, according to the Principles of the Declaration of Helsinki. Every study member was informed about the procedure and the study and signed an informed consent.

**Fig. 1:**
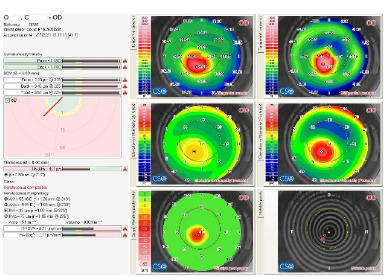
Tomography report of a 34-year-old patient with keratoconus on the right eye

The study compared uncorrected visual acuity (UCVA) and best corrected visual acuity (BCVA) at pre-operative and 6-month post-operative consults. The measurement system used Snellen eyechart, with standardized decimal, as required by the European norm (EN ISO 8596, previously DIN 58220). The tomography parameters were obtained from the corneal tomography device Sirius® (Scheimpflug tomography system - CSO Inc.), which can generate a keratoconus report (like the one in **Fig. 1**).

We could compare central corneal thickness (CCT) and the thinnest corneal thickness (TCT) before intervention and after 6 months. Maximal corneal curvature, abbreviated with K_max_, was also analyzed. Based on the Scheimpflug tomography system, we also analyzed the symmetry index of curvature. It is defined as the difference of mean anterior tangential curvature (in diopters) of two circular zones centered on the vertical axis in inferior and superior hemispheres [7]. The two circular zones are centered in x = 0 mm, y = ±1.5 mm, and their radius is 1.5 mm [7]. The symmetry index of the front cornea (Sif) and posterior cornea (Sib) are expressed in diopters, measuring the vertical asymmetry [7]. In our case, the positive values indicated that the inferior hemisphere was steeper than the superior one. Other parameters available were the anterior and posterior keratoconus vertex (KVf and KVb), and they defined the highest point of ectasia on anterior and posterior elevation maps of anterior and posterior corneal surfaces, respectively [7].

Baiocchi-Calossi-Versaci front and back index (BCVf) and (BCVb) evaluate the presence of an ectasia through the analysis of the coma and trefoil components of Zernike’s decomposition of elevations in the zones where keratoconus statistically arises [7]. Based on the presumption that ectasia statistically develops in a preferential direction (inferotemporal), it mainly manifests in coma, trefoil, and spherical aberration [7].

Both the keratoconus vertex front and back and the Baiocchi-Calossi-Versaci index front and back were analyzed before and after 6 months of intervention.

### Surgical technique

**Fig. 2:**
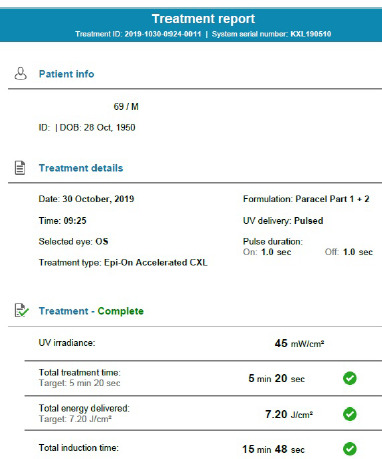
Treatment report generated at the end of the A-CXL procedure

The study was based on accelerated epithelium-on collagen crosslinking utilizing the Avedro KXL 190510 system. After instilling Oxybuprocaine hydrochloride 4 mg/ml 3 times every 5 minutes in the operating room, under sterile conditions, the instillation of 0.25% Riboflavin HPMC, BAC, EDTA TRIS solution (DOC: Paracel solution). The higher concentration of Riboflavin is specially designed for the epi-on method, allowing faster penetration and diffusion into the corneal stroma. After this instillation, the cornea is exposed to UV irradiance for 5 minutes and 20 seconds at a 45 mW/cm^2^ power, obtaining a total of 7.20 J/cm^2^ energy delivery. During this time, Paracel solution part 2 is still instilled every 15 seconds. The protocol consists of pulsed UV delivery (1:1), as shown in the treatment report (**Fig. 2**).

**Fig. 3:**
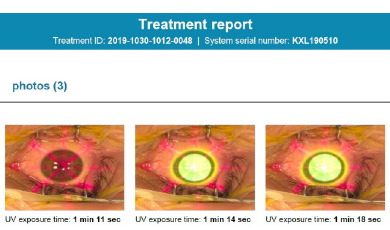
The first picture is taken during the pulse-off period, and the others during the pulse-on phase of the treatment

The device also allows pictures of the eye being treated during the procedure (**Fig. 3**). After the eye is washed with BSS (20 ml balanced salt solution) solution and moxifloxacin drops, a bandage contact lens is placed.

### Statistics analysis

The study uses Microsoft Excel, the Social Science Statistics website, and Med Calc statistical software to group, sort, correlate, and analyze case data. We used descriptive statistical methods like mean, maximum, minimum, standard deviation, and median. The significance level was assessed at p < 0.05.

## Results

**Fig. 4:**
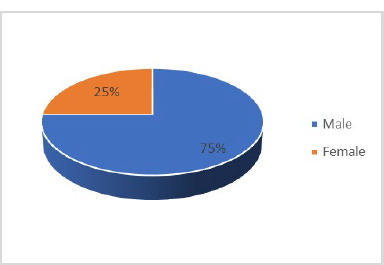
Sex distribution in the studying group

Ten patients had monocular interventions, and another ten had binocular procedures, resulting in 30 eyes being treated. The distribution on sex ratio was 1:3, with only five females being treated, compared to 15 males (shown in **Fig. 4**).

**Fig. 5:**
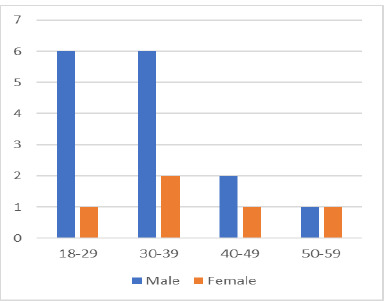
Age distribution over the decades for both male and female

At the time of initial examination, the mean age of the patients treated was 33.5 ± 9 years old. **Fig. 5** analyzes the age distribution of the treated patients, both male and female, with numerous cases being consulted in the specific age gap for keratoconus (the second and third decades of life).

### Pachymetry comparisons

When comparing central corneal thickness (CCT), we obtain a mean value of 480.8 ± 33.23 µm pre-surgery and 451.23 ± 29.25 µm during the 6-month check-up. With a significance level of 0.0006, we could conclude that the decrease in central cornea was relevant in the follow-up of the A-CXL patients. Another parameter monitored in the topography readings was the thinnest corneal thickness (TCT). The mean value registered for it was 466.43 ± 31.24 µm before the intervention and 438,63 ±3,53 µm after the crosslinking procedure. Obtaining a p-value of 0,0009, the evolution of this measurement was statistically relevant. More collected data is available in **Table 1**.

**Table 1:** Pachymetry data before surgery and after 6 months

	Mean	SD	Std Error Mean
CCT preop.	480.8	33.235	6.067
CCT 6 mo.	451.23	29.254	5.341
TCT preop.	466.433	31.24	5.703
TCT 6 mo.	48.63	30.539	5.575

### Evolution of the spherical and cylinder diopters throughout the treatment

**Fig. 6:**
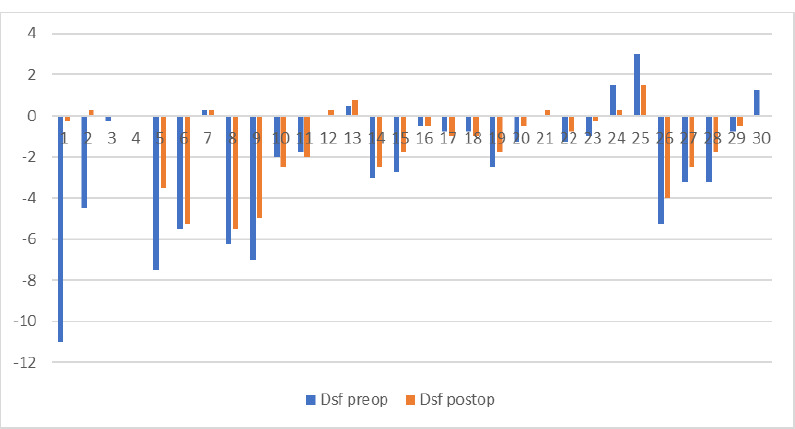
The correlation between spherical diopters before and 6 months after A-CXL

**Fig. 7:**
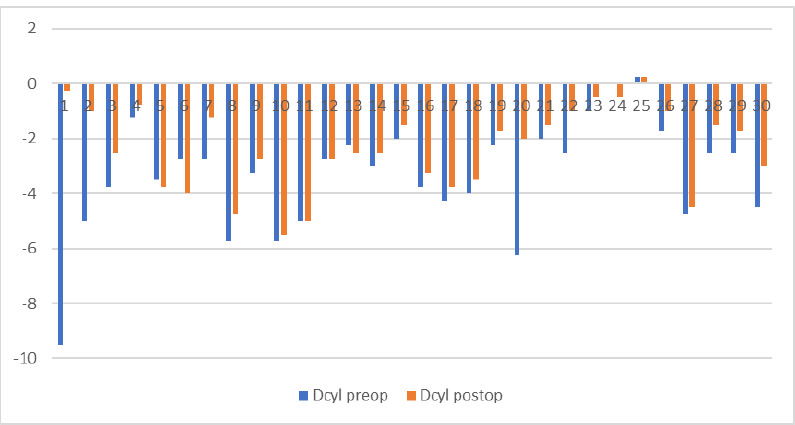
The correlation between cylinder diopters before and 6 months after A-CXL

The data obtained from autorefractometry showed spherical diopters between -11.00 and +3.00, with a mean value of -2.18 ± 3.05, and cylinder diopters between -9.25 and +0.25, with a mean value of -3.33 ± 1.98 pre-operatory. At the six-month check-up, the minimum spherical diopters were -5.50, and the maximum spherical diopters were +1.50, with a mean value of -1.30 ± 1.84. The cylinder measurements were between -5.50 and +0.25, with an average value of -2.33 ± 1.52. The correlation between these values is presented in **Fig. 6** and **7**.

### Evaluation of the visual acuity with and without correction

**Fig. 8:**
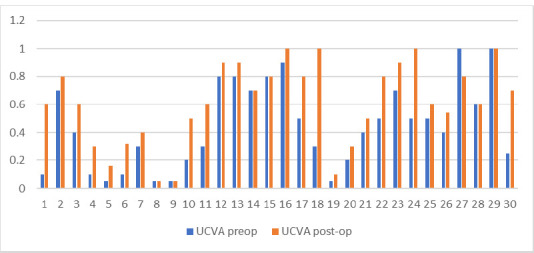
UCVA before and after Accelerated epi-on Crosslinking

**Fig. 9:**
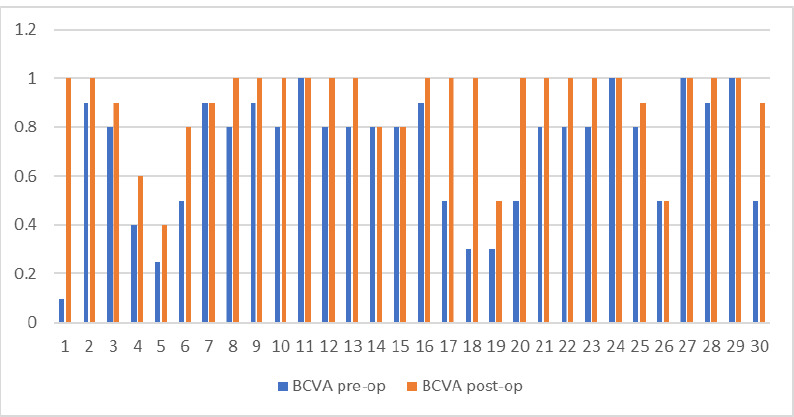
BCVA before and after Accelerated epi-on Crosslinking

There was a good correlation between uncorrected visual acuity (UCVA) before the surgery and after 6 months. The minimum and the maximum values were the same, ranging from 0.05 to 1, but the mean value increased from 0.441 ± 0.30 to 0.61 ± 0.29. The p-value was 0.0319, so it had statistical relevance. The better outcome was the one regarding the best corrected visual acuity (BCVA). In this parameter, we could observe a growth in minimum value from 0.1 to 0.4, with the maximum remaining the same, but the mean value also improved after treatment. We measured an average of 0.705 ± 0.252 before the A-CXL and a mean of 0.9 ± 0.174 after 6 months. The significance level was high, with a p-value of 0.0009. More information is shown in **Fig. 8, 9**.

### Evolution of topography indices associated with keratoconus screening

As mentioned above, we considered Kmax, the Symmetry index of curvature front and back (SIf and SIb), the Anterior and Posterior Keratoconus Vertex (KVf and KVb), and the Baiocchi-Calossi-Versaci front and back index (BCVf and BCVb). The differences between measurements made before surgery and after 6 months were relatively low and were not statistically significant, as shown in **Table 2**.

**Table 2:** Comparison of topography indices

			Paired differences	
		95% Confidence interval		
	Mean	Lower	Upper	p
SIf preop.-SIf postop.	-0.01	-1.5011	1.4812	0.9894
SIb preop.-SIb postop.	0.031	-0.3407	0.402	0.8693
KVf preop.-KVf postop.	-0.267	-6.7304	6.197	0.9345
KVb preop.-KVb postop.	2.003	-13.689	17.6947	0.7992
BCVf preop.-BCVf postop.	0.017	-0.6711	0.7045	0.9614
BCVb preop.-BCVb postop.	0.125	-0.596	0.8464	0.7295
K_max_ preop.-K_max_ postop.	-1.143	-2.988	0.7020	0.2199

## Discussions

Every study finds a higher frequency of keratoconus diagnosis among men, and ours met this tendency. With only 25% of the patients treated being women, the ratio was 1:3, comparable with most studies observing keratoconus patients. The age distribution could also be framed in the general tendency. We already know that keratoconus is a disease of young people, affecting mainly patients between 20 and 40 years of age. The average age at the beginning of the study was 33.5 ± 9 years old.

This study showed that pachymetry measurements like CCT and TCT are statistically relevant in keratoconus patients who were followed up at 6 months. The minimum and maximum values were lower after 6 months, and the mean values were correlated with reduced thickness.

The evolution of spherical and cylindrical diopters 6 months after the accelerated epi-on Crosslinking showed a remarkable diminishment. On average, after using the procedure, almost one diopter was reduced.

Visual acuity was also a good marker for follow-up six months after surgery. Both uncorrected visual acuity (UCVA) and best corrected visual acuity (BCVA) showed improvement at the half-year follow-up.

Regarding the specific topography markers, like SIf/SIb, KVf/KVb, BCVf/BCVb, and K_max_, minor differences were observed at 6 months follow-up and were not considered statistically relevant. Other studies show a better correlation at 1-year check-ups and similar results at 6-month follow-ups [8].

## Conclusion

Based on the data obtained in this study, we can advocate for the importance of long-term follow-up for patients who underwent A-CXL surgery. Although some of the measured parameters show statistically relevant alteration at 6 months post-operatory, like pachymetry values, visual acuity, and refractometry values, others have a lower rate of change, being reliable after one year, like the markers analyzed over corneal topography (K_max_, SIf/SIb, KVf/KVb, BCVf/BCVb).
